# A triclinic polymorph of miconazole

**DOI:** 10.1107/S2056989024000276

**Published:** 2024-01-26

**Authors:** Hanna Kaspiaruk, Lilianna Chęcińska

**Affiliations:** a University of Lodz Doctoral School of Exact and Natural Sciences, Narutowicza 68, 90-136 Łódź, Poland; b University of Lodz, Faculty of Chemistry, Pomorska 163/165, 90-236 Łódź, Poland; Texas A & M University, USA

**Keywords:** miconazole, crystal structure, Hirshfeld surface, energy frameworks

## Abstract

The crystal structure of the new triclinic polymorph of miconazole {MIC; C_18_H_14_Cl_4_N_2_O; systematic name: (*RS*)-1-[2-(2,4-di­chloro­benz­yloxy)-2-(2,4-di­chloro­phen­yl)eth­yl]-1*H*-imidazole} is reported and compared with the monoclinic form of solvent-free miconazole previously reported [Kaspiaruk & Chęcińska (2022 ▸). *Acta Cryst.* C**78**, 343–350]. A comparison shows a different orientation of imidazole and one di­chloro­phenyl ring between polymorphic mol­ecules. In the crystal structure of the title compound, only weak halogen bonds and C—H⋯π(arene) inter­actions are found. Hirshfeld surface analysis and energy framework calculations complement the comparison of the two polymorphic forms of the miconazole drug.

## Abstract 2

A second polymorphic form of solvent-free miconazole, a triclinic form (MIC-tri), is reported, and compared with the anhydrous monoclinic form of miconazole (MIC-mono).

## 1. Chemical context

Miconazole {MIC; C_18_H_14_Cl_4_N_2_O; CAS No. 22916-47-8; systematic name: (*RS*)-1-[2-(2,4-di­chloro­benz­yloxy)-2-(2,4-di­chloro­phen­yl)eth­yl]-1*H*-imidazole} is a drug that belongs to the group of first-generation imidazole derivatives. It shows a broad spectrum of anti­fungal activity against dermatophytes, yeasts, and Gram-positive bacteria (Botter, 1971 ▸; Sawyer *et al.*, 1975 ▸; Nenoff *et al.*, 2017 ▸). Miconazole exhibits poor aqueous solubility, therefore salts (Peeters *et al.*, 2004 ▸; Patel *et al.*, 2018 ▸), co-crystals (Drozd *et al.*, 2021 ▸, 2022 ▸) and mol­ecular salts (Drozd *et al.*, 2021 ▸) with this agent have been synthesized to improve its bioavailability.

The first crystal structure of miconazole in the form of a hemihydrate was published previously (Peeters *et al.*, 1979 ▸). A monoclinic anhydrous form and solvatomorphs, namely hemi-hydrogen peroxide solvate, monohydrate, ethanol monosolvate and methanol monosolvate, have been published recently (Kersten *et al.*, 2018 ▸; Kaspiaruk & Chęcińska, 2022 ▸; Panini *et al.*, 2022 ▸).

In this article a second polymorphic form of pure solvent-free miconazole, a triclinic form (MIC-tri), is reported, and compared with the anhydrous monoclinic form of miconazole (MIC-mono) (Kaspiaruk & Chęcińska, 2022 ▸; Panini *et al.*, 2022 ▸). 
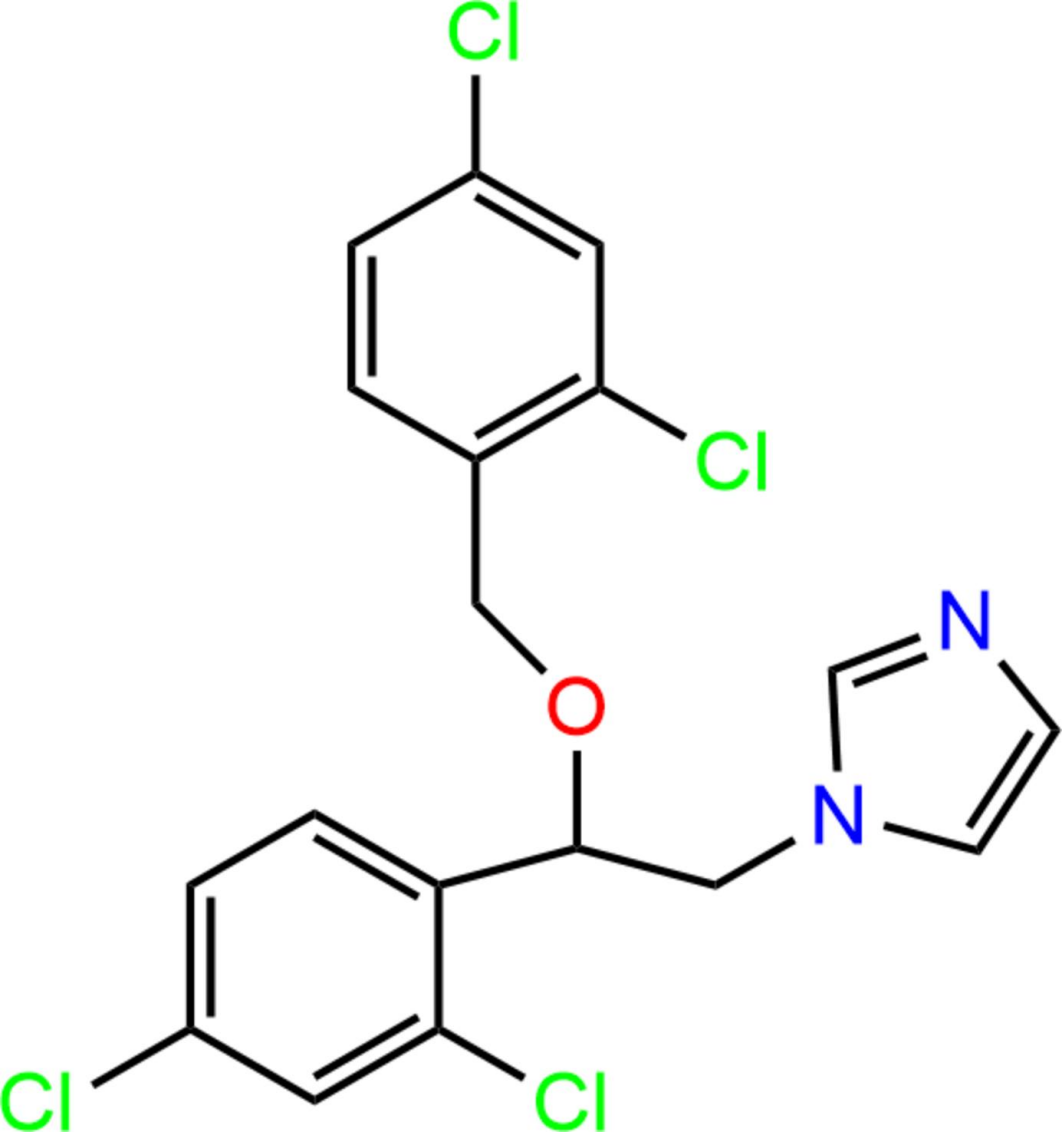


## 2. Structural commentary

**Figure 1:**
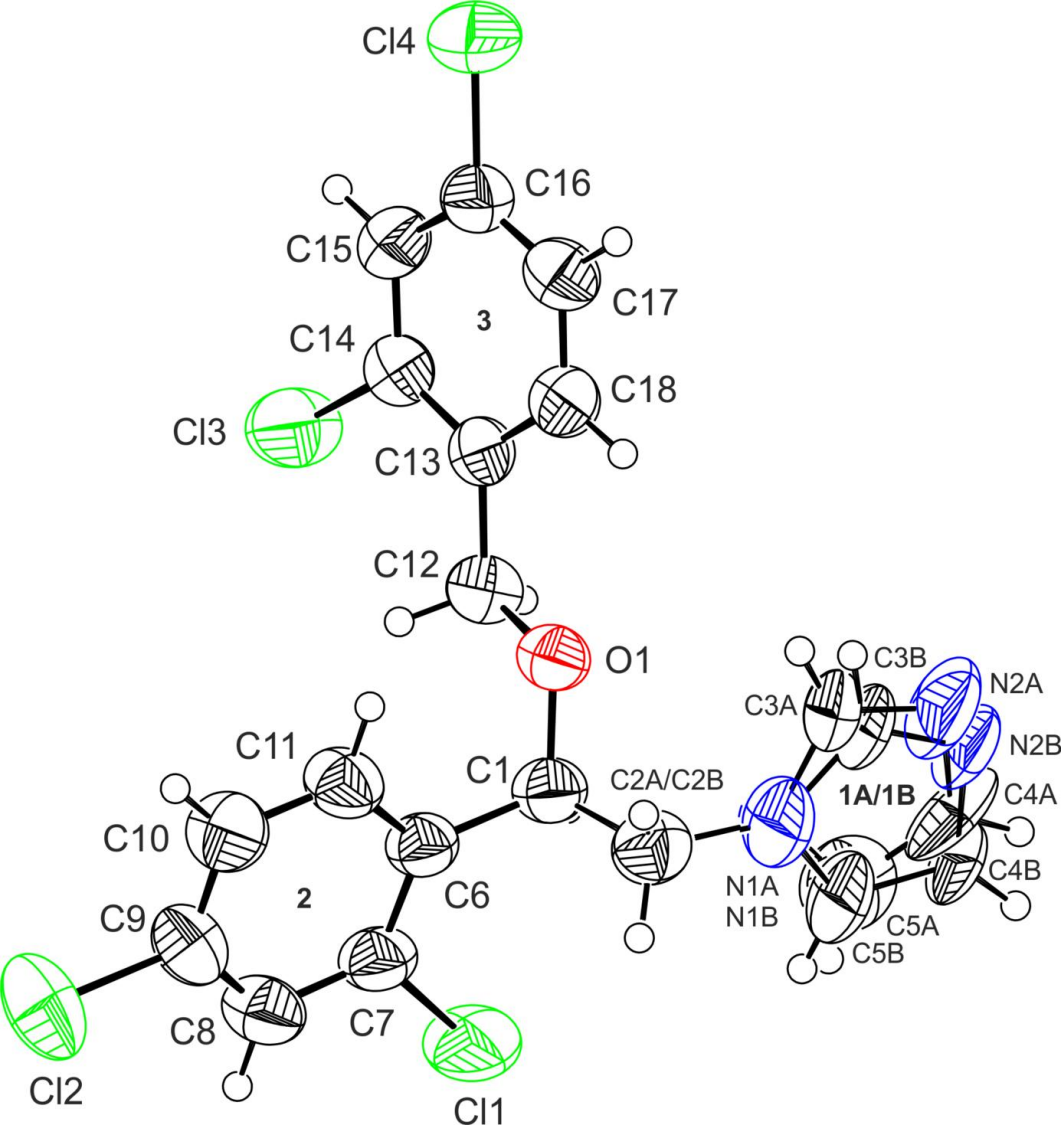
The mol­ecular structure of MIC-tri showing the atom-labelling scheme. The disorder components *A* and *B* have equal site-occupancies (1/2). Labels **1A**, **1B**, **2** and **3** refer to the best planes of the aromatic rings. Displacement ellipsoids are drawn at the 50% probability level.

The mol­ecular structure of the title compound (MIC-tri) is illustrated in Fig. 1 ▸. It crystallizes in the triclinic crystal system, space group *P* 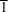 . The mol­ecule of miconazole consists of three planar groups: an imidazole ring (ring 1) and two di­chloro­phenyl groups (ring 2, atoms C6–C11; ring 3, atoms C13–C18) connected by a flexible meth­oxy­ethyl fragment. In the MIC-tri structure, the imidazole ring was found to be disordered over two orientations (ring 1*A*: N1*A*, C3*A*, N2*A*, C4*A*, C5*A* and ring 1*B*: N1*B*, C3*B*, N2*B*, C4*B*, C5*B*, respectively) with equal occupancies (0.5).

**Figure 2:**
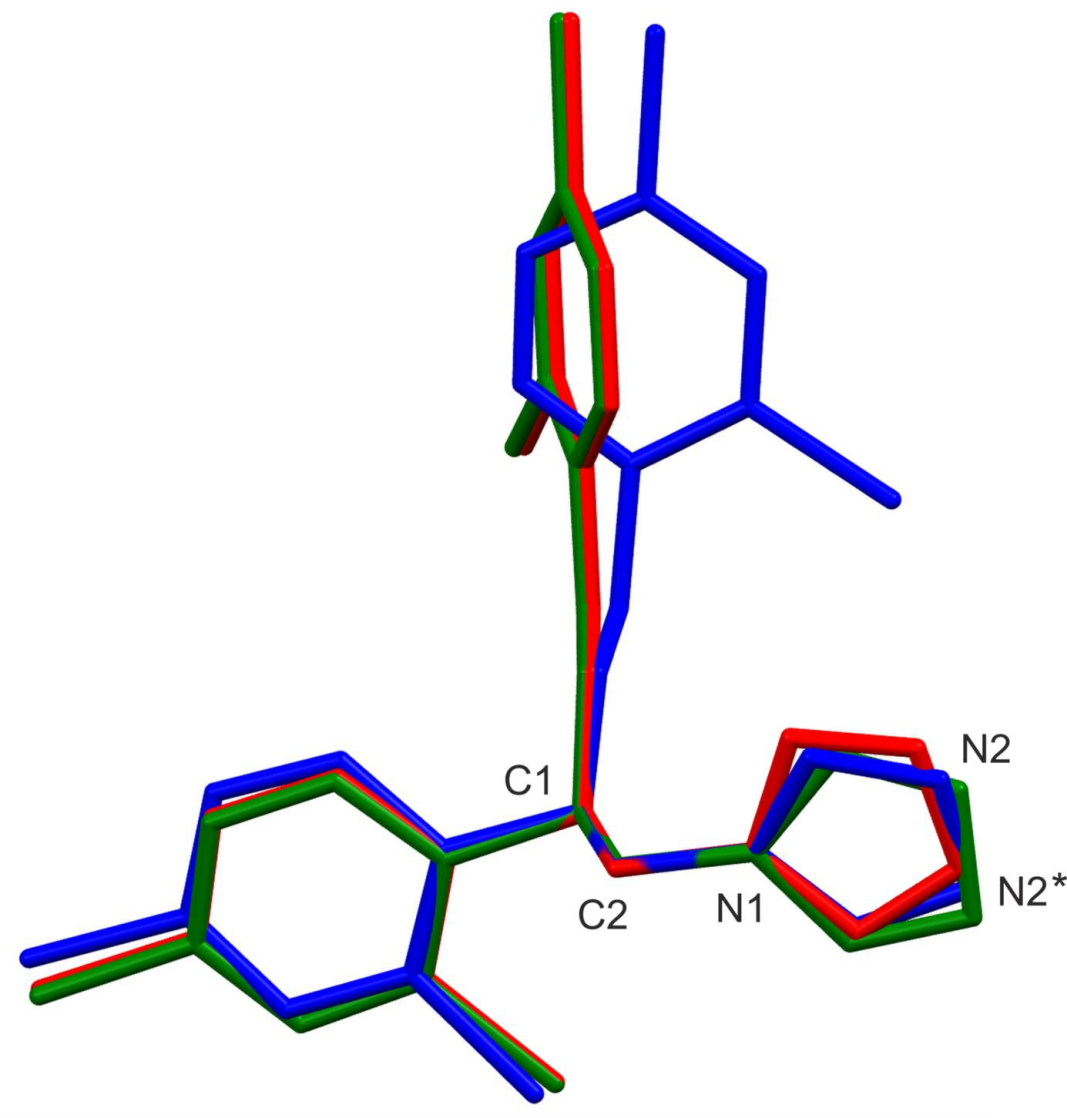
An overlay of three miconazole mol­ecules, showing the best fit for atoms C1, C2 and N1: the colour code is blue = MIC-mono, red = MIC-tri-*A*, green = MIC-tri-*B*. N2* is the position of the N2 atom in mol­ecule MIC-mono.

To make a comparison between the triclinic and monoclinic polymorphic forms of miconazole, the superposition of the three miconazole skeletons is shown in Fig. 2 ▸, considering separately two disorder components *A* and *B* of MIC-tri (MIC-tri-*A* and MIC-tri-*B*). One can see the difference in the orientation of the di­chloro­phenyl ring (ring 3) in the two polymorphic forms: they are approximately perpendicular to each other. Inter­estingly, such an orientation of the arene ring (ring 3) as observed in the MIC-tri form seems to be preferable for hydrated/solvated forms of miconazole (Kaspiaruk & Chęcińska, 2022 ▸). Additionally, the compared polymorphs also differ from each other with regard to the position of the N2 atom of the imidazole ring in that they are related by a rotation of about 180°. The mutual arrangement of the aromatic rings in the analysed miconazole mol­ecules can be described by the dihedral angles between their best planes, calculated by the least-squares method (Table 1 ▸).

## 3. Supra­molecular features

**Figure 3:**
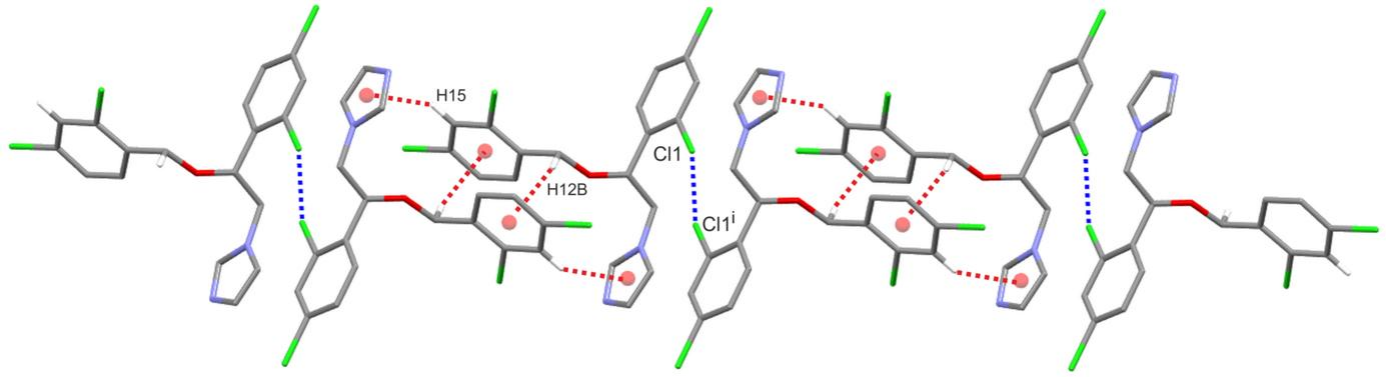
A part of the crystal structure of MIC-tri (only disorder component *A* is shown) showing the formation of C7—Cl1⋯Cl1(−*x*, −*y*, 1 − *z*) halogen bonds and C—H⋯π(arene) inter­actions between adjacent mol­ecules. Red balls represent the centroids of the phenyl rings (*Cg*1*A* and *Cg*3). Inter­actions are shown as dashed lines (blue and red), and for the sake of clarity, H atoms not involved in these inter­actions have been omitted.

In the crystal structure of the title miconazole polymorph (MIC-tri), there are no typical hydrogen bonds. In contrast to the monoclinic form (MIC-mono), where two C—H⋯*X* (*X* = N, Cl) inter­actions were observed, here only a weak C7—Cl1⋯Cl1(−*x*, −*y*, 1 − *z*) halogen inter­action is found (Fig. 3 ▸); the distance of the close Cl1⋯Cl1 contact is 3.250 (3) Å and the C7—Cl1⋯Cl1 angle is 162.92 (2)°. Close inspection of the crystal packing of MIC-tri also reveals two C—H⋯π(arene) inter­actions: C12—H12*B*⋯*Cg*3(−*x*, 1 − *y*, −*z*) [H⋯*Cg*3 = 2.78 Å, C—H⋯*Cg*3 = 151°] and C15—H15⋯*Cg*1*A*/1*B*(−*x*, 1 − *y*, −*z*) [H⋯*Cg*1*A*/1*B* = 2.96 Å/2.94 Å, C—H⋯*Cg*1*A*/1*B* = 140°/145°] (Fig. 3 ▸).

## 4. Hirshfeld surface analysis

**Figure 4:**
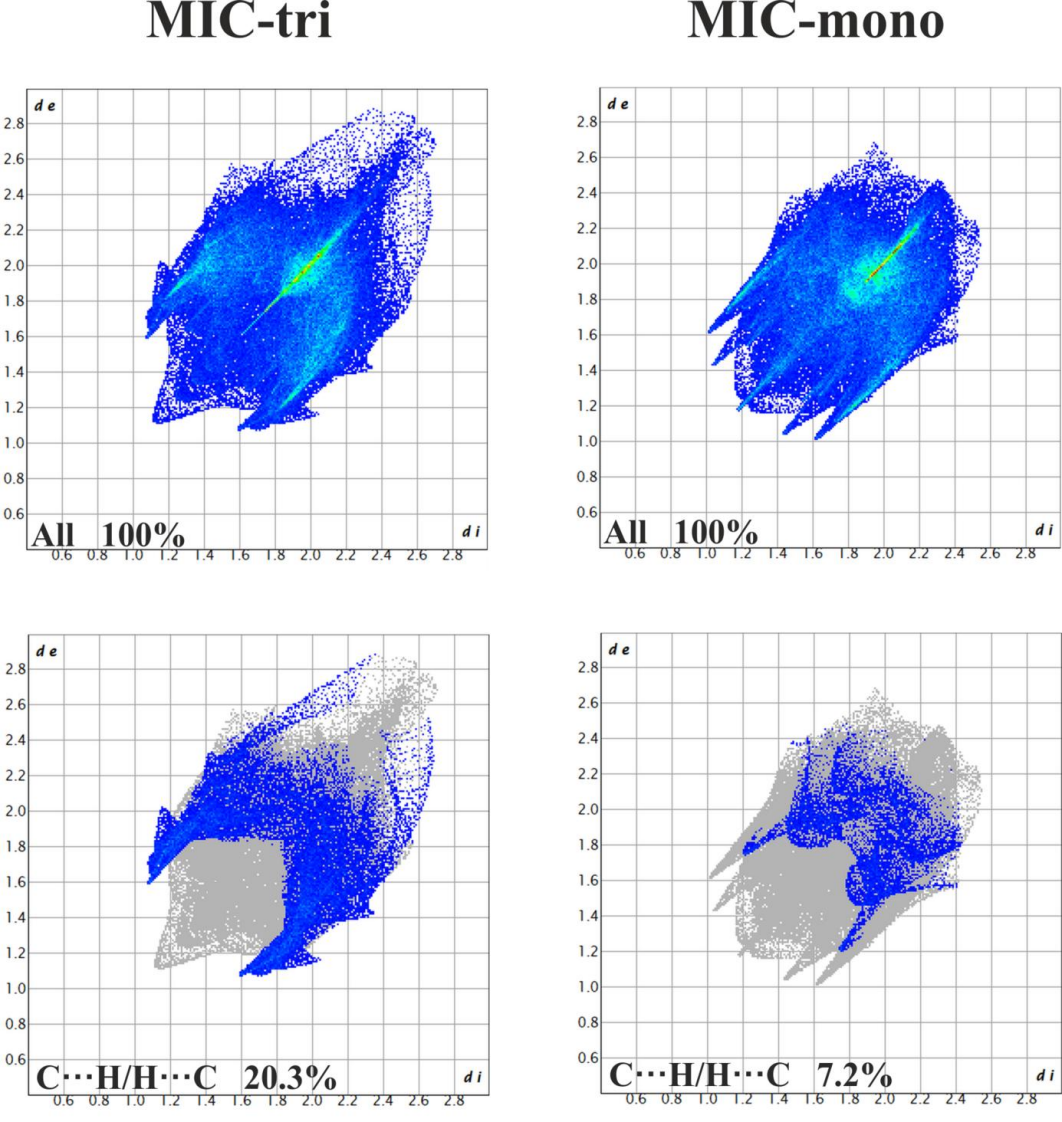
Comparison of two-dimensional fingerprint plots for the two miconazole polymorphic forms, MIC-tri (disorder component *A* only) and MIC-mono, showing all close contacts, and delineated into C⋯H/H⋯C inter­actions. The *d*_i_ and *d*_e_ values are the closest inter­nal and external distances (in Å) from given points on the Hirshfeld surface.

**Figure 5:**
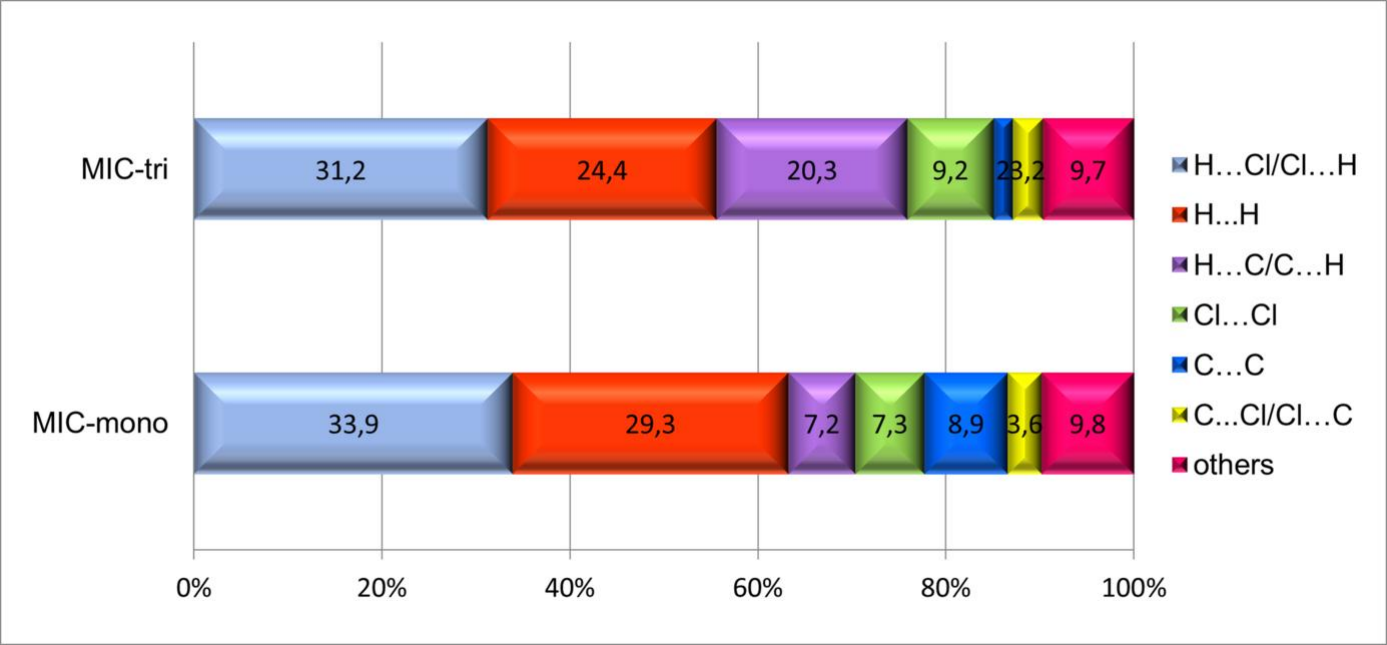
Diagram showing the percentage contributions of different close contacts to the Hirshfeld surface area of miconazole mol­ecules in the two polymorphic forms, MIC-tri (disorder component *A* only) and MIC-mono.

Hirshfeld surface analysis (Spackman & Jayatilaka, 2009 ▸) complements the comparison of the two polymorphic forms of the miconazole drug. Hirshfeld surfaces and fingerprint plots (Spackman & McKinnon, 2002 ▸; Spackman & Jayatilaka, 2009 ▸) were generated using *CrystalExplorer* software (Turner *et al.*, 2017 ▸). Fig. 4 ▸ presents the 2D fingerprint plots of all close contacts characteristic of the MIC-tri and MIC-mono structures; among them, additionally, C⋯H/H⋯C inter­actions are highlighted as their contributions to the Hirshfeld surface differ the most between polymorphs. An increase in the number of such inter­actions is observed for MIC-tri. As shown in breakdown diagrams (Fig. 5 ▸), in contrast to the C⋯H inter­actions, the contribution of C⋯C close contacts, mainly representing aromatic π–π inter­actions, decreased quite significantly for the triclinic form (2.0%) compared to the monoclinic one (9.8%). Other close contacts remain essentially comparable; the H⋯Cl/Cl⋯H and H⋯H contacts have the largest share of the Hirshfeld surface of both analysed polymorphs.

## 5. Pairwise model energies and their energy frameworks

The similarities and differences between two polymorphic forms of miconazole can also be analysed by comparison of the inter­action energies calculated between mol­ecules within a representative cluster of 3.8 Å from the crystal lattices and their visualization as energy frameworks. All inter­action energies for MIC-tri are listed in Table 2 ▸. Similarly to the analysis presented previously for MIC-mono (Kaspiaruk & Chęcińska, 2022 ▸), only one mol­ecular pair has a relatively high total energy value over 50 kJ mol^−1^ with the highest calculated contribution of dispersive and repulsive forces resulting from C—H⋯π(arene) inter­actions (Fig. 3 ▸). Unfortunately, it is quite difficult to assign the remaining energies from the table to specific inter­actions in the crystal of MIC-tri because of the limited number of contacts that met the geometrical criteria of hydrogen bonds. For example, the total energy value of the mol­ecular pair connected by the Cl1⋯Cl1 halogen bond is only −6.6 kJ mol^−1^ while much higher total energies (45.0, 35.4, 25.5, 23.4, in kJ mol^−1^) seem to result from the specific mutual arrangement of mol­ecules supported by the weaker aromatic π–π inter­actions.

Generally, for MIC-tri, the contribution of dispersive forces predominates over electrostatic ones. The relationship between these two forces can be expressed by the proportions of electrostatic (Σ*kE*_ele_) and dispersion (Σ*kE*_dis_) energies (both scaled), given as percentages, that contribute to their sums for all mol­ecular pairs in the cluster of mol­ecules Σ*kE*_ele_/(Σ*kE*_ele_+Σ*kE*_dis_); [Σ*kE*_dis_/(Σ*kE*_dis_+Σ*kE*_ele_)]. The percentages showing the proportion of the electrostatic component to the dispersion component are: 20%:80% for MIC-tri, which is comparable to MIC-mono (26%:74%).

**Figure 6:**
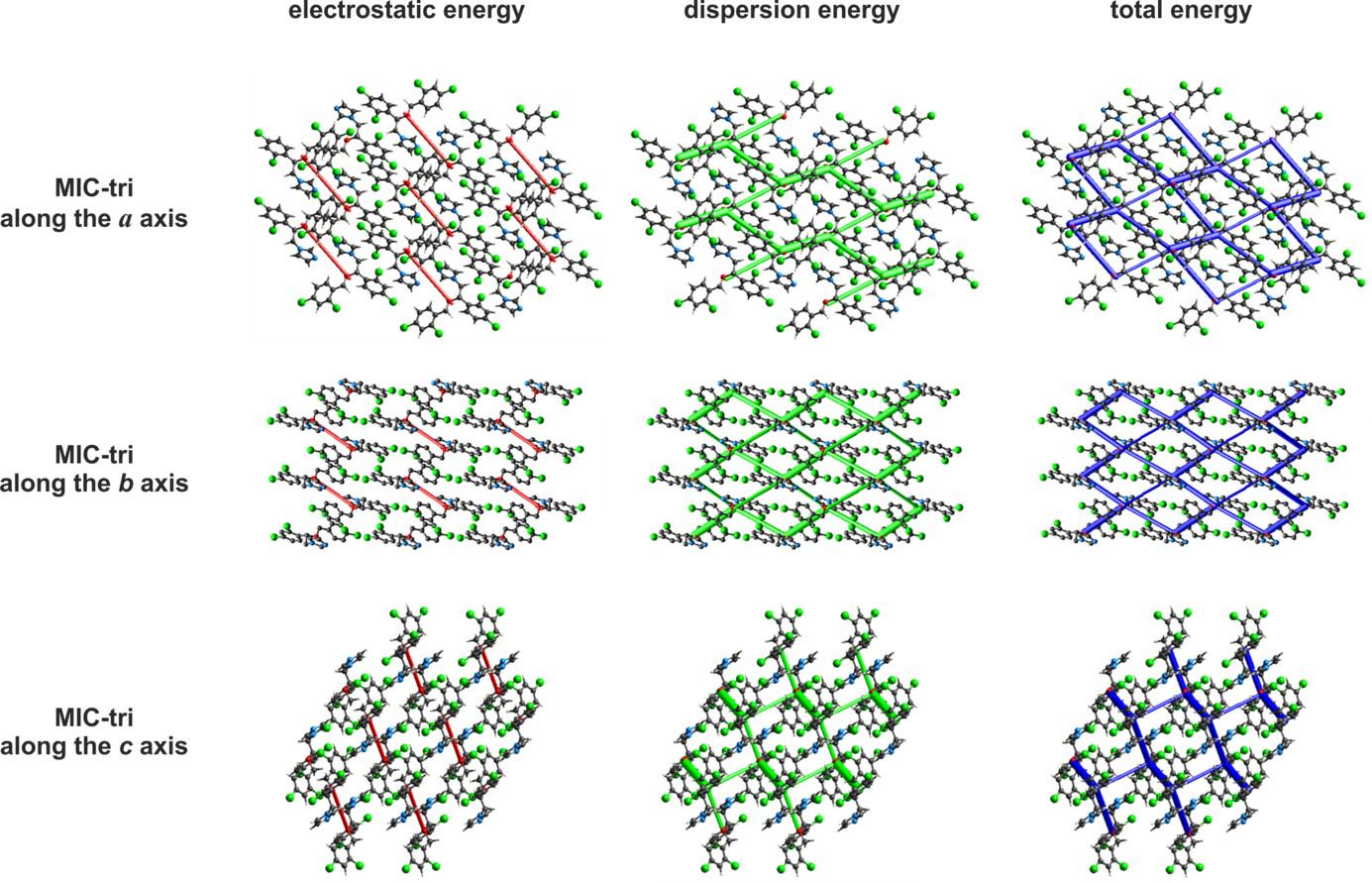
Representative energy framework diagrams for separate electrostatic (red) and dispersion (green) components, and the total inter­action energy (blue) for MIC-tri (disorder component *A* only). All diagrams use the same energy tube scale factor of 80 and an energy threshold of 20 kJ mol^−1^ to be compatible with Fig. 6 ▸ (Kaspiaruk & Chęcińska, 2022 ▸).

Fig. 6 ▸ shows the representative energy frameworks for the analysed structure of MIC-tri. Energies between two mol­ecules are represented as cylinders connecting these mol­ecular pairs, with the radius of the cylinder proportional to the contribution of the corresponding energy. Red individual cylinders correspond to electrostatic energy (*E*_ele_), green to dispersive energy (*E*_dis_), and blue to total energy (*E*_tot_). Views along all crystallographic axes demonstrate that the MIC-tri structure exhibits a tri-periodic energy pattern; the total energy framework reflects the framework of its dominant dispersion component. Pairwise model energies (Turner *et al.*, 2014 ▸) were estimated and visualized (Turner *et al.*, 2015 ▸; Mackenzie *et al.*, 2017 ▸) between mol­ecules within a cluster with a radius of 3.8 Å, using *CrystalExplorer* software (Turner *et al.*, 2017 ▸). The computational approach uses a B3LYP/6-31G(d,p) mol­ecular wave function calculated for the respective mol­ecular arrangement in the crystal. The total inter­action energy between any nearest-neighbour mol­ecular pairs was estimated in terms of four components: electrostatic, polarization, dispersion and exchange-repulsion, with scale factors (*k*) of 1.057, 0.740, 0.871 and 0.618, respectively.

## 6. Database survey

A search of the Cambridge Structural Database (CSD version 5.44, September 2023, Groom *et al.*, 2016 ▸) revealed only one solvent-free miconazole form in the monoclinic system (PAVPIP; Panini *et al.*, 2022 ▸; PAVPIP01; Kaspiaruk & Chęcińska, 2022 ▸).

## 7. Synthesis and crystallization

A second polymorphic form of solvent-free miconazole (MIC-tri) was found after a couple of months, probably as an effect of decomposition of miconazole co-crystals with small aromatic carb­oxy­lic acids or any other hydrated/solvated forms of miconazole. The MIC-tri crystals are dull and yellow in colour; they are distinctly different from the co-crystals (Fig. S1 in the supporting information).

## 8. Refinement

Crystal data, data collection and structure refinement details are summarized in Table 3 ▸. During the refinement of the title compound MIC-tri, the imidazole ring was found to be disordered over two orientations (ring 1*A*: N1*A*, C3*A*, N2*A*, C4*A*, C5*A* and ring 1*B*: N1*B*, C3*B*, N2*B*, C4*B*, C5*B*); site occupancies of two components were fixed at 0.5. Component *B* of the disordered imidazole ring was restrained using RIGU and SADI commands in *SHELXL*. Furthermore, the C2 methyl­ene atom was also split; constraints (EXYZ and EADP) were used to fix the overlapping atoms C2*A* and C2*B*. It was difficult to determine the position of the nitro­gen N2 atom in the disordered imidazole ring, mainly due to the poor quality of the crystals for which the single-crystal diffraction pattern was disturbed by powder diffraction effects.

All hydrogen atoms bonded to carbon atoms were placed geometrically and refined as riding, with *U*_iso_(H) = 1.2*U*_eq_(C) for the methyl­ene, methine and aromatic groups.

## Supplementary Material

Crystal structure: contains datablock(s) I, global. DOI: 10.1107/S2056989024000276/jy2040sup1.cif

Structure factors: contains datablock(s) I. DOI: 10.1107/S2056989024000276/jy2040Isup2.hkl

The MIC-tri crystals. DOI: 10.1107/S2056989024000276/jy2040sup3.tif

Supporting information file. DOI: 10.1107/S2056989024000276/jy2040Isup4.cml

CCDC reference: 2324176

Additional supporting information:  crystallographic information; 3D view; checkCIF report

## Figures and Tables

**Table 1:** Dihedral angles (°) between the best planes in pure solvent-free polymorphic MIC-structures **1**(*A*/*B*) is the imidazole ring, **2** and **3** are the di­chloro­phenyl rings.

	**1**(*A*/*B*)/**2**	**1**(*A*/*B*)/**3**	**2**/**3**
MIC-tri-*A*	2.4 (1)	68.5 (1)	69.2 (3)
MIC-tri-*B*	1.5 (1)	68.0 (1)	69.2 (3)
MIC-mono	16.8 (2)	22.2 (2)	5.4 (2)

**Table 2:** Inter­action energies (kJ mol^−1^) for the cluster of mol­ecules with a radius of 3.8 Å for MIC-tri *N* is the number of mol­ecular pairs. *R* is the distance (Å) between mol­ecular centroids. *E*_tot_ is the total energy and *E*_ele_ is the electrostatic (*k* = 1.057), *E*_pol_ is the polarization (*k* = 0.740), *E*_dis_ is the dispersion (*k* = 0.871) and *E*_rep_ is the repulsion (*k* = 0.618) component.

*N*	*R*	*kE*_ele_	*kE*_pol_	*kE*_dis_	*kE*_rep_	*E*_tot_
1	10.41	−4.65	−0.44	−11.15	9.64	−6.6
2	9.42	−2.01	−1.26	−19.77	7.66	−15.3
1	6.73	−16.28	−3.26	−64.11	27.01	−56.6
1	7.88	−8.46	−0.67	−43.29	17.00	−35.4
2	9.49	−0.21	−0.44	−7.40	4.02	−4.1
1	10.72	−1.27	−0.15	−11.67	3.77	−9.2
1	9.90	−27.38	−5.77	−19.25	7.54	−45.0
1	8.58	3.59	−1.26	−32.49	6.86	−23.4
2	14.04	−2.85	−0.07	−4.88	4.51	−3.3
2	13.03	−2.01	−0.07	−6.62	3.58	−5.1
1	8.22	−6.76	−0.96	−26.91	9.08	−25.5
1	10.23	−3.38	−0.74	−11.93	5.99	−10.1

**Table 3:** Experimental details

Crystal data	
Chemical formula	C_18_H_14_Cl_4_N_2_O
*M*_r_	416.11
Crystal system, space group	Triclinic, *P* 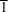
Temperature (K)	295
*a*, *b*, *c* (Å)	8.8691 (8), 9.4161 (9), 13.0347 (11)
α, β, γ (°)	75.502 (8), 85.013 (8), 62.478 (10)
*V* (Å^3^)	934.11 (17)
*Z*	2
Radiation type	Cu *K*α
μ (mm^−1^)	5.83
Crystal size (mm)	0.26 × 0.09 × 0.05

Data collection	
Diffractometer	XtaLAB Synergy, Dualflex, HyPix
Absorption correction	Gaussian (*CrysAlis PRO*; Rigaku OD, 2023 ▸)
*T*_min_, *T*_max_	0.521, 1.000
No. of measured, independent and observed [*I* > 2σ(*I*)] reflections	8698, 3412, 2548
*R*_int_	0.051
(sin θ/λ)_max_ (Å^−1^)	0.610

Refinement	
*R*[*F* ^2^ > 2σ(*F* ^2^)], *wR*(*F* ^2^), *S*	0.085, 0.224, 1.10
No. of reflections	3412
No. of parameters	271
No. of restraints	35
H-atom treatment	H-atom parameters constrained
Δρ_max_, Δρ_min_ (e Å^−3^)	0.47, −0.34

Computer programs: *CrysAlis PRO* (Rigaku OD, 2023 ▸), *SHELXT* (Sheldrick, 2015*a* ▸), *SHELXL2019/2* (Sheldrick, 2015*b* ▸), *Mercury* (Macrae *et al.*, 2020 ▸), *PLATON* (Spek, 2020 ▸) and *publCIF* (Westrip, 2010 ▸).

## supplementary crystallographic information

### Crystal data

**Table:**

C_18_H_14_Cl_4_N_2_O	*Z* = 2
*M**_r_* = 416.11	*F*(000) = 424
Triclinic, *P*1	*D*_x_ = 1.479 Mg m^−^^3^
*a* = 8.8691 (8) Å	Cu *K*α radiation, λ = 1.54184 Å
*b* = 9.4161 (9) Å	Cell parameters from 3917 reflections
*c* = 13.0347 (11) Å	θ = 5.4–69.1°
α = 75.502 (8)°	µ = 5.83 mm^−^^1^
β = 85.013 (8)°	*T* = 295 K
γ = 62.478 (10)°	Prism, pale yellow
*V* = 934.11 (17) Å^3^	0.26 × 0.09 × 0.05 mm

### Data collection

**Table:**

XtaLAB Synergy, Dualflex, HyPix diffractometer	3412 independent reflections
Radiation source: micro-focus sealed X-ray tube, PhotonJet (Cu) X-ray Source	2548 reflections with *I* > 2σ(*I*)
Mirror monochromator	*R*_int_ = 0.051
Detector resolution: 10.0000 pixels mm^-1^	θ_max_ = 70.0°, θ_min_ = 3.5°
ω scans	*h* = −10→8
Absorption correction: gaussian (CrysAlisPro; Rigaku OD, 2023)	*k* = −11→10
*T*_min_ = 0.521, *T*_max_ = 1.000	*l* = −15→15
8698 measured reflections	

### Refinement

**Table:**

Refinement on *F*^2^	Primary atom site location: structure-invariant direct methods
Least-squares matrix: full	Hydrogen site location: inferred from neighbouring sites
*R*[*F*^2^ > 2σ(*F*^2^)] = 0.085	H-atom parameters constrained
*wR*(*F*^2^) = 0.224	*w* = 1/[σ^2^(*F*_o_^2^) + (0.0932*P*)^2^ + 1.192*P*] where *P* = (*F*_o_^2^ + 2*F*_c_^2^)/3
*S* = 1.10	(Δ/σ)_max_ < 0.001
3412 reflections	Δρ_max_ = 0.47 e Å^−^^3^
271 parameters	Δρ_min_ = −0.34 e Å^−^^3^
35 restraints	

### Special details

**Table:**

Geometry. All esds (except the esd in the dihedral angle between two l.s. planes) are estimated using the full covariance matrix. The cell esds are taken into account individually in the estimation of esds in distances, angles and torsion angles; correlations between esds in cell parameters are only used when they are defined by crystal symmetry. An approximate (isotropic) treatment of cell esds is used for estimating esds involving l.s. planes.

### Fractional atomic coordinates and isotropic or equivalent isotropic displacement parameters (Å^2^)

**Table:**

	*x*	*y*	*z*	*U*_iso_*/*U*_eq_	Occ. (<1)
Cl1	0.0819 (3)	0.1162 (2)	0.51579 (13)	0.1083 (7)	
Cl2	0.2681 (2)	0.4980 (3)	0.65792 (14)	0.1076 (6)	
Cl3	−0.1424 (2)	0.80046 (19)	0.10302 (13)	0.1010 (6)	
Cl4	0.2330 (2)	0.8416 (2)	−0.23760 (12)	0.0995 (6)	
O1	0.2576 (4)	0.2922 (4)	0.2041 (2)	0.0625 (8)	
C1	0.2397 (6)	0.1992 (6)	0.3050 (4)	0.0607 (11)	
H1	0.131505	0.194421	0.305888	0.073*	
C2A	0.3861 (7)	0.0273 (7)	0.3190 (4)	0.0746 (14)	0.5
H2A1	0.491611	0.034582	0.303657	0.090*	0.5
H2A2	0.393547	−0.032985	0.392160	0.090*	0.5
N1A	0.365 (2)	−0.0616 (18)	0.2502 (11)	0.065 (6)	0.5
C3A	0.431 (3)	−0.047 (3)	0.1510 (13)	0.073 (5)	0.5
H3A	0.486108	0.015526	0.121529	0.087*	0.5
N2A	0.399 (3)	−0.140 (2)	0.1066 (17)	0.108 (5)	0.5
C4A	0.303 (2)	−0.206 (2)	0.178 (2)	0.098 (9)	0.5
H4A	0.260498	−0.272593	0.163709	0.117*	0.5
C5A	0.285 (4)	−0.159 (4)	0.268 (3)	0.116 (14)	0.5
H5A	0.230134	−0.185213	0.328805	0.140*	0.5
C2B	0.3861 (7)	0.0273 (7)	0.3190 (4)	0.0746 (14)	0.5
H2B1	0.491111	0.035528	0.303531	0.090*	0.5
H2B2	0.393827	−0.031688	0.392517	0.090*	0.5
N1B	0.370 (3)	−0.067 (2)	0.2518 (15)	0.091 (8)	0.5
C3B	0.405 (3)	−0.083 (3)	0.1500 (17)	0.075 (5)	0.5
H3B	0.459801	−0.030432	0.103672	0.091*	0.5
N2B	0.353 (2)	−0.1802 (18)	0.1247 (12)	0.075 (4)	0.5
C4B	0.281 (3)	−0.243 (2)	0.2120 (15)	0.072 (4)	0.5
H4B	0.240982	−0.320628	0.219884	0.087*	0.5
C5B	0.286 (4)	−0.162 (4)	0.281 (2)	0.089 (9)	0.5
H5B	0.234048	−0.168814	0.346279	0.106*	0.5
C6	0.2468 (6)	0.2745 (6)	0.3939 (3)	0.0579 (11)	
C7	0.1771 (7)	0.2453 (6)	0.4925 (4)	0.0666 (13)	
C8	0.1827 (7)	0.3111 (7)	0.5737 (4)	0.0726 (14)	
H8	0.135273	0.288707	0.638479	0.087*	
C9	0.2605 (7)	0.4110 (7)	0.5565 (4)	0.0724 (14)	
C10	0.3307 (7)	0.4456 (7)	0.4610 (4)	0.0771 (15)	
H10	0.382023	0.514534	0.450659	0.093*	
C11	0.3238 (7)	0.3763 (7)	0.3805 (4)	0.0688 (13)	
H11	0.371934	0.398643	0.316069	0.083*	
C12	0.1108 (6)	0.4420 (6)	0.1695 (4)	0.0614 (11)	
H12A	0.078220	0.504246	0.223656	0.074*	
H12B	0.017817	0.420131	0.157645	0.074*	
C13	0.1438 (6)	0.5415 (5)	0.0680 (3)	0.0528 (10)	
C14	0.0343 (6)	0.7054 (6)	0.0308 (4)	0.0624 (12)	
C15	0.0596 (7)	0.8006 (6)	−0.0624 (4)	0.0680 (13)	
H15	−0.016094	0.911639	−0.086078	0.082*	
C16	0.2007 (7)	0.7245 (6)	−0.1184 (4)	0.0657 (12)	
C17	0.3117 (7)	0.5628 (7)	−0.0858 (4)	0.0661 (12)	
H17	0.405896	0.514221	−0.125471	0.079*	
C18	0.2828 (6)	0.4704 (6)	0.0077 (4)	0.0626 (12)	
H18	0.358039	0.359034	0.030281	0.075*	

### Atomic displacement parameters (Å^2^)

**Table:**

	*U*^11^	*U*^22^	*U*^33^	*U*^12^	*U*^13^	*U*^23^
Cl1	0.1649 (17)	0.1257 (13)	0.0818 (10)	−0.1152 (14)	0.0240 (10)	−0.0122 (9)
Cl2	0.1128 (13)	0.1450 (16)	0.0939 (11)	−0.0657 (12)	0.0058 (9)	−0.0633 (11)
Cl3	0.0908 (11)	0.0796 (9)	0.0871 (10)	−0.0107 (8)	0.0275 (8)	−0.0090 (7)
Cl4	0.1312 (14)	0.0926 (11)	0.0754 (9)	−0.0619 (10)	0.0262 (9)	−0.0070 (8)
O1	0.069 (2)	0.0617 (18)	0.0508 (16)	−0.0262 (16)	0.0056 (14)	−0.0107 (14)
C1	0.068 (3)	0.063 (3)	0.054 (2)	−0.035 (2)	0.001 (2)	−0.007 (2)
C2A	0.080 (4)	0.067 (3)	0.070 (3)	−0.031 (3)	−0.006 (3)	−0.009 (3)
N1A	0.049 (8)	0.047 (8)	0.062 (7)	0.006 (5)	0.000 (5)	−0.009 (6)
C3A	0.099 (12)	0.053 (9)	0.079 (8)	−0.040 (7)	0.008 (7)	−0.029 (6)
N2A	0.142 (14)	0.064 (10)	0.140 (13)	−0.062 (8)	−0.015 (10)	−0.026 (8)
C4A	0.093 (13)	0.046 (10)	0.16 (3)	−0.052 (8)	0.012 (14)	0.001 (12)
C5A	0.098 (19)	0.09 (2)	0.127 (19)	−0.023 (15)	0.039 (16)	−0.028 (15)
C2B	0.080 (4)	0.067 (3)	0.070 (3)	−0.031 (3)	−0.006 (3)	−0.009 (3)
N1B	0.127 (16)	0.085 (13)	0.098 (10)	−0.075 (12)	0.009 (9)	−0.035 (8)
C3B	0.105 (10)	0.055 (10)	0.095 (8)	−0.056 (8)	0.005 (7)	−0.027 (6)
N2B	0.091 (10)	0.044 (7)	0.101 (8)	−0.040 (6)	−0.013 (6)	−0.013 (6)
C4B	0.102 (9)	0.047 (8)	0.095 (8)	−0.054 (7)	0.002 (7)	−0.021 (7)
C5B	0.12 (2)	0.070 (15)	0.109 (9)	−0.069 (16)	0.006 (9)	−0.033 (9)
C6	0.066 (3)	0.060 (3)	0.053 (2)	−0.036 (2)	0.001 (2)	−0.007 (2)
C7	0.083 (3)	0.068 (3)	0.057 (3)	−0.047 (3)	0.003 (2)	−0.004 (2)
C8	0.079 (4)	0.087 (4)	0.055 (3)	−0.045 (3)	0.008 (2)	−0.012 (2)
C9	0.072 (3)	0.088 (4)	0.063 (3)	−0.037 (3)	0.000 (2)	−0.027 (3)
C10	0.088 (4)	0.092 (4)	0.077 (3)	−0.063 (3)	0.001 (3)	−0.019 (3)
C11	0.078 (3)	0.086 (3)	0.058 (3)	−0.052 (3)	0.012 (2)	−0.017 (2)
C12	0.059 (3)	0.066 (3)	0.056 (2)	−0.027 (2)	0.002 (2)	−0.010 (2)
C13	0.052 (3)	0.056 (2)	0.054 (2)	−0.026 (2)	0.0002 (19)	−0.0151 (19)
C14	0.065 (3)	0.062 (3)	0.059 (3)	−0.028 (2)	0.006 (2)	−0.017 (2)
C15	0.074 (3)	0.053 (3)	0.069 (3)	−0.024 (2)	0.002 (2)	−0.010 (2)
C16	0.081 (3)	0.067 (3)	0.057 (3)	−0.041 (3)	0.005 (2)	−0.013 (2)
C17	0.065 (3)	0.076 (3)	0.059 (3)	−0.034 (3)	0.010 (2)	−0.018 (2)
C18	0.064 (3)	0.058 (3)	0.061 (3)	−0.023 (2)	0.001 (2)	−0.014 (2)

### Geometric parameters (Å, º)

**Table:**

Cl1—C7	1.733 (5)	C3B—H3B	0.9300
Cl2—C9	1.741 (5)	N2B—C4B	1.391 (17)
Cl3—C14	1.736 (5)	C4B—C5B	1.335 (15)
Cl4—C16	1.752 (5)	C4B—H4B	0.9300
O1—C12	1.405 (6)	C5B—H5B	0.9300
O1—C1	1.428 (5)	C6—C11	1.383 (7)
C1—C2B	1.514 (7)	C6—C7	1.397 (7)
C1—C2A	1.514 (7)	C7—C8	1.368 (7)
C1—C6	1.522 (6)	C8—C9	1.370 (8)
C1—H1	0.9800	C8—H8	0.9300
C2A—N1A	1.444 (12)	C9—C10	1.376 (8)
C2A—H2A1	0.9700	C10—C11	1.387 (7)
C2A—H2A2	0.9700	C10—H10	0.9300
N1A—C5A	1.367 (16)	C11—H11	0.9300
N1A—C3A	1.372 (13)	C12—C13	1.511 (6)
C3A—N2A	1.30 (2)	C12—H12A	0.9700
C3A—H3A	0.9300	C12—H12B	0.9700
N2A—C4A	1.421 (18)	C13—C14	1.372 (6)
C4A—C5A	1.331 (17)	C13—C18	1.384 (6)
C4A—H4A	0.9300	C14—C15	1.387 (7)
C5A—H5A	0.9300	C15—C16	1.374 (7)
C2B—N1B	1.453 (12)	C15—H15	0.9300
C2B—H2B1	0.9700	C16—C17	1.353 (7)
C2B—H2B2	0.9700	C17—C18	1.389 (7)
N1B—C3B	1.364 (15)	C17—H17	0.9300
N1B—C5B	1.376 (14)	C18—H18	0.9300
C3B—N2B	1.32 (2)		
			
C12—O1—C1	112.8 (3)	C4B—C5B—H5B	121.6
O1—C1—C2B	106.6 (4)	N1B—C5B—H5B	121.6
O1—C1—C2A	106.6 (4)	C11—C6—C7	116.6 (4)
O1—C1—C6	111.2 (4)	C11—C6—C1	121.1 (4)
C2B—C1—C6	109.8 (4)	C7—C6—C1	122.4 (4)
C2A—C1—C6	109.8 (4)	C8—C7—C6	123.3 (5)
O1—C1—H1	109.7	C8—C7—Cl1	117.5 (4)
C2A—C1—H1	109.7	C6—C7—Cl1	119.1 (4)
C6—C1—H1	109.7	C7—C8—C9	117.9 (5)
N1A—C2A—C1	111.7 (7)	C7—C8—H8	121.1
N1A—C2A—H2A1	109.3	C9—C8—H8	121.1
C1—C2A—H2A1	109.3	C8—C9—C10	121.7 (5)
N1A—C2A—H2A2	109.3	C8—C9—Cl2	119.0 (4)
C1—C2A—H2A2	109.3	C10—C9—Cl2	119.3 (4)
H2A1—C2A—H2A2	107.9	C9—C10—C11	119.0 (5)
C5A—N1A—C3A	113.0 (16)	C9—C10—H10	120.5
C5A—N1A—C2A	129.2 (15)	C11—C10—H10	120.5
C3A—N1A—C2A	117.8 (15)	C6—C11—C10	121.5 (5)
N2A—C3A—N1A	105.7 (16)	C6—C11—H11	119.3
N2A—C3A—H3A	127.1	C10—C11—H11	119.3
N1A—C3A—H3A	127.1	O1—C12—C13	110.1 (4)
C3A—N2A—C4A	108.0 (16)	O1—C12—H12A	109.6
C5A—C4A—N2A	110.3 (18)	C13—C12—H12A	109.6
C5A—C4A—H4A	124.9	O1—C12—H12B	109.6
N2A—C4A—H4A	124.9	C13—C12—H12B	109.6
C4A—C5A—N1A	103 (2)	H12A—C12—H12B	108.2
C4A—C5A—H5A	128.5	C14—C13—C18	117.6 (4)
N1A—C5A—H5A	128.5	C14—C13—C12	120.9 (4)
N1B—C2B—C1	113.7 (10)	C18—C13—C12	121.5 (4)
N1B—C2B—H2B1	108.8	C13—C14—C15	122.6 (5)
C1—C2B—H2B1	108.8	C13—C14—Cl3	119.4 (4)
N1B—C2B—H2B2	108.8	C15—C14—Cl3	117.9 (4)
C1—C2B—H2B2	108.8	C16—C15—C14	117.4 (4)
H2B1—C2B—H2B2	107.7	C16—C15—H15	121.3
C3B—N1B—C5B	99.6 (14)	C14—C15—H15	121.3
C3B—N1B—C2B	136.9 (16)	C17—C16—C15	122.3 (5)
C5B—N1B—C2B	123.1 (14)	C17—C16—Cl4	119.6 (4)
N2B—C3B—N1B	112.5 (14)	C15—C16—Cl4	118.2 (4)
N2B—C3B—H3B	123.8	C16—C17—C18	119.0 (5)
N1B—C3B—H3B	123.8	C16—C17—H17	120.5
C3B—N2B—C4B	109.8 (13)	C18—C17—H17	120.5
C5B—C4B—N2B	100.9 (14)	C13—C18—C17	121.1 (4)
C5B—C4B—H4B	129.6	C13—C18—H18	119.5
N2B—C4B—H4B	129.6	C17—C18—H18	119.5
C4B—C5B—N1B	116.9 (17)		
			
C12—O1—C1—C2B	166.2 (4)	C2A—C1—C6—C7	−84.5 (6)
C12—O1—C1—C2A	166.2 (4)	C11—C6—C7—C8	−0.2 (8)
C12—O1—C1—C6	−74.1 (5)	C1—C6—C7—C8	179.7 (5)
O1—C1—C2A—N1A	−71.4 (9)	C11—C6—C7—Cl1	−178.9 (4)
C6—C1—C2A—N1A	168.0 (9)	C1—C6—C7—Cl1	1.0 (7)
C1—C2A—N1A—C5A	−89 (3)	C6—C7—C8—C9	0.2 (8)
C1—C2A—N1A—C3A	89.4 (16)	Cl1—C7—C8—C9	178.9 (4)
C5A—N1A—C3A—N2A	−3 (3)	C7—C8—C9—C10	0.2 (8)
C2A—N1A—C3A—N2A	178.8 (14)	C7—C8—C9—Cl2	179.5 (4)
N1A—C3A—N2A—C4A	3 (2)	C8—C9—C10—C11	−0.6 (9)
C3A—N2A—C4A—C5A	−3 (3)	Cl2—C9—C10—C11	−179.8 (4)
N2A—C4A—C5A—N1A	1 (3)	C7—C6—C11—C10	−0.1 (8)
C3A—N1A—C5A—C4A	1 (3)	C1—C6—C11—C10	179.9 (5)
C2A—N1A—C5A—C4A	179.2 (15)	C9—C10—C11—C6	0.5 (9)
O1—C1—C2B—N1B	−71.8 (10)	C1—O1—C12—C13	174.5 (4)
C6—C1—C2B—N1B	167.6 (9)	O1—C12—C13—C14	−165.8 (4)
C1—C2B—N1B—C3B	84 (3)	O1—C12—C13—C18	15.8 (6)
C1—C2B—N1B—C5B	−88 (3)	C18—C13—C14—C15	−1.1 (7)
C5B—N1B—C3B—N2B	−2 (3)	C12—C13—C14—C15	−179.5 (5)
C2B—N1B—C3B—N2B	−175 (2)	C18—C13—C14—Cl3	179.8 (4)
N1B—C3B—N2B—C4B	−2 (3)	C12—C13—C14—Cl3	1.3 (6)
C3B—N2B—C4B—C5B	5 (3)	C13—C14—C15—C16	0.2 (8)
N2B—C4B—C5B—N1B	−7 (4)	Cl3—C14—C15—C16	179.4 (4)
C3B—N1B—C5B—C4B	6 (4)	C14—C15—C16—C17	0.5 (8)
C2B—N1B—C5B—C4B	−180 (2)	C14—C15—C16—Cl4	178.9 (4)
O1—C1—C6—C11	−22.3 (6)	C15—C16—C17—C18	−0.4 (8)
C2B—C1—C6—C11	95.4 (5)	Cl4—C16—C17—C18	−178.7 (4)
C2A—C1—C6—C11	95.4 (5)	C14—C13—C18—C17	1.2 (7)
O1—C1—C6—C7	157.7 (4)	C12—C13—C18—C17	179.7 (4)
C2B—C1—C6—C7	−84.5 (6)	C16—C17—C18—C13	−0.5 (8)
