# Supplementary figures and images for: A triclinic polymorph of miconazole
Source: Acta Crystallogr E Crystallogr Commun. 2024 Jan 26;80(Pt 2):196–200. doi: 10.1107/S2056989024000276 (PMC10848966; doi:10.1107/S2056989024000276)

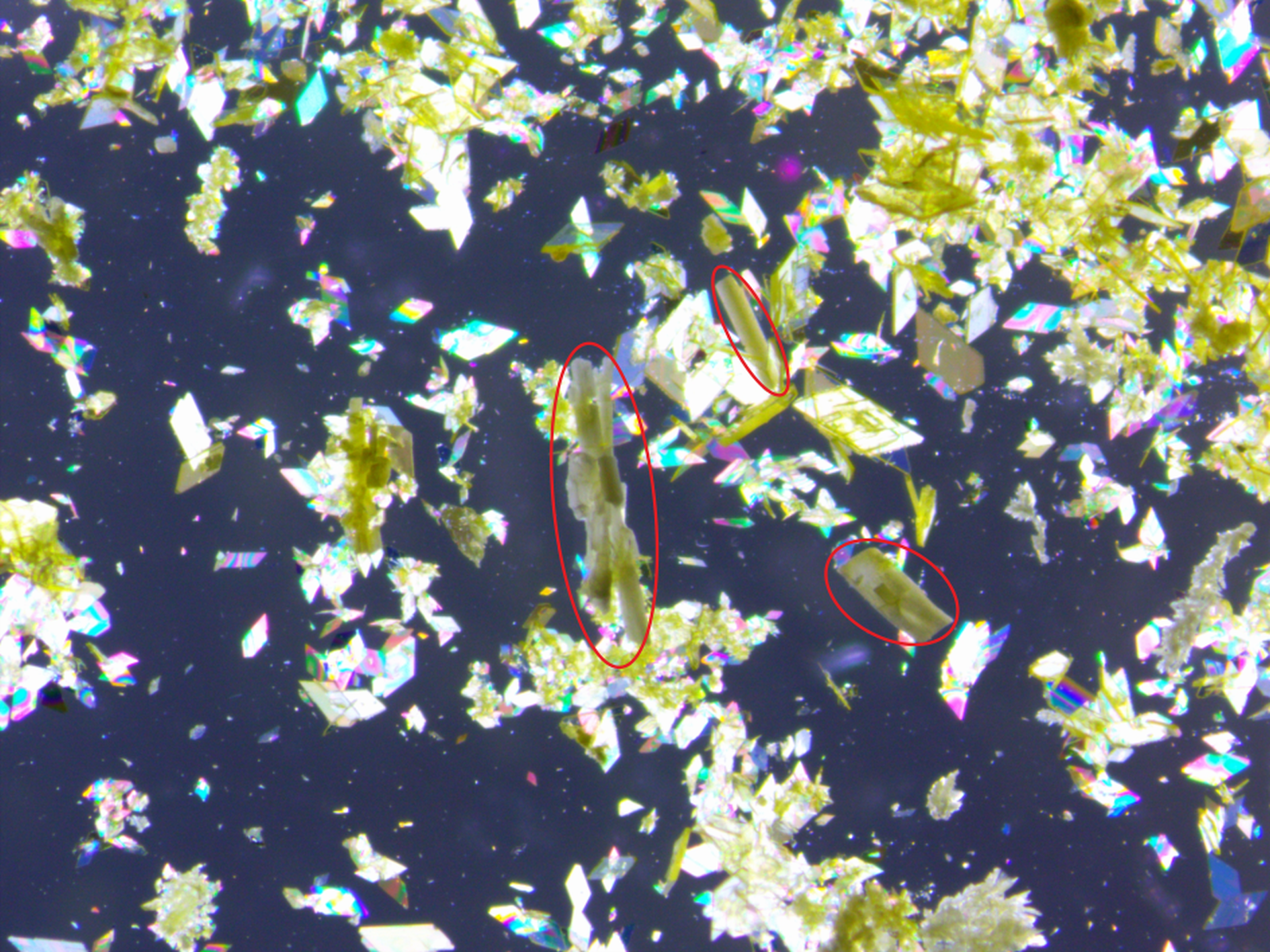

Supplement: Supplementary file 3 [file e-80-00196-sup3.tif]
